# Colorimetric Sensor Based on *β*-Cyclodextrin-Functionalized Silver Nanoparticles for Zidovudine Sensitive Determination

**DOI:** 10.1155/2020/5054864

**Published:** 2020-06-17

**Authors:** Farhad Ghanizadeh Gerayeli, Farnaz Hosseini, Zohreh Bagheri, Amir Savardashtaki, Zahra Shabaninejad, Ali Mohammad Amani, Sohrab Najafipour

**Affiliations:** ^1^Department of Medical Nanotechnology, School of Advanced Medical Sciences and Technologies, Shiraz University of Medical Sciences, Shiraz, Iran; ^2^Department of Physiology, School of Medicine, Shiraz University of Medical Sciences, Shiraz, Iran; ^3^Department of Medical Biotechnology, School of Advanced Medical Sciences and Technologies, Shiraz University of Medical Sciences, Shiraz, Iran; ^4^Department of Nanotechnology, Faculty of Biological Sciences, Tarbiat Modares University, Tehran, Iran; ^5^Pharmaceutical Sciences Research Center, Shiraz University of Medical Sciences, Shiraz, Iran; ^6^Department of Microbiology, School of Medicine, Fasa University of Medical Sciences, Fasa, Iran; ^7^Non Communicable Diseases Research Center, School of Medicine, Fasa University of Medical Sciences, Fasa, Iran

## Abstract

Zidovudine (ZDV) is an antiviral drug against HIV that was approved by the FDA on March, 1987. It is a reverse transcriptase inhibitor. This type of drug stops the reproduction of DNA and decreases the amount of the virus in the patients' blood. Due to the ability of forming various molecular bonds, silver nanoparticles (AgNPs) are widely used for the detection of large range of agents, including drugs. In this study, we synthesized AgNP-modified *β*-cyclodextrin (*β*-CD) using green synthesis for the sensitive and selective zidovudine (ZDV) determination. Characterization of nanoparticles was done using different methods including infrared (IR) spectroscopy, ultraviolet-visible (UV-Vis) spectroscopy, transmission electron microscopy (TEM), and also, X-ray diffraction (XRD) patterns. The AgNP *λ*-max peak was at approximately 405 nm. In the presence of ZDV, yellow solution was turned to red color, and surface plasmon absorption band was dramatically centered at 560 nm. ZDV was determined in the range of 50–500 *μ*M, and the detection limit value was obtained as 42 *μ*M. The sensor was used to determine ZDV in tablets with good recovery.

## 1. Introduction

**Scheme 1:**
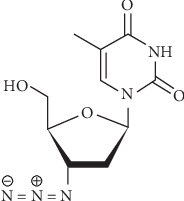
Structure of ZDV.

Zidovudine (ZDV), also known as 3-azido-3-deoxythymidine, is a chemical component which can be phosphorylated to ZDV triphosphate in the body. The research studies indicated that phosphorylate form could prevent HIV replication with inhibition of the reverse transcriptase as a key enzyme. Moreover, ZDV is the first approved drug for AIDS and HIV disease treatment. The chemical structure of ZDV is shown in Scheme 1 [1].

Biosensors are defined as analytical devices which can detect analytes quantitatively or semiquantitatively by using biological components such as enzymes, aptamers, and antibodies as recognition elements [2]. Several methods have been used to measure ZDV, including electrochemical methods [3], and also, high-performance liquid chromatography (HPLC) [4], but the colorimetric method using AgNPs has not been reported for this compound yet. Because of excellent plasmon absorption properties of novel metal nanoparticles (NPs), especially gold nanoparticles (AuNPs) [5] and AgNPs [6], a variety of colorimetric sensors have been developed [7, 8]. It is a sensor by which the detection of various analytes could be recognized with naked eyes [8, 9]. The key point of designing a colorimetric sensor is how an organic compound can be used as a modifier for this metal and the author's knowledge on how to interact analytes with the sensor by considering all possibilities for interactions between a sensor and another compound. Various compounds including amino acids [10], chitosan [11], and citrate [12] have been used to repair these metals, such as gold and silver. Various compounds including amino acids [13], metal ions [10], and proteins [11] have been measured by using different types of nanoparticles of noble metal sensors. Due to unique properties including antibacterial and thermal activity, surface plasmon resonance (SPR), and catalytic and electronic properties, AgNPs have been applied remarkably in various fields [12, 14–18]. Some other materials such as chitosan [19] and cyclodextrin (CD) [20] have been used to attach silver. CDs are cyclic oligosaccharides consisting of glucopyranosyl units linked by *α*-(1,4) bonds; their unique structures consist of a hydrophobic cavity and a hydrophilic surface and can form a complex structure with different groups [21]. In addition to improving solubility of insoluble compounds, CD can improve the stabilization of guests against oxidation, volatility, and sublimation, and also, mask off-flavors and control drug release to modify drug taste [22]. Due to its availability and inexpensiveness, *β*-cyclodextrin is available, inexpensive, and most applicable CD with 200–800 Da molecular weight [23]. Therefore, *β*-cyclodextrin is used as a stabilizer for silver nanoparticles. In this research, we presented a simple, easy, and inexpensive optical chemosensor for ZDV based on silver nanoparticles.

In this research, we proposed a sensitive detection of ZDV using the AgNP SPR property. Addition of ZDV caused a diminished SPR intensity and a red shift in synthetized AgNPs. Moreover, color of AgNP solution has been changed by adding different ZDV concentrations.

## 2. Materials and Methods

### 2.1. Materials

We purchased silver nitrate (AgNO3) and *β*-cyclodextrin and Chitosan from Merck and Sigma-Aldrich, respectively. Milli-Q purified water was applied for sample preparations. After appropriate dilution, we used these solutions for the spectroscopic studies. We used hydrochloric acid (0.1 M) and sodium hydroxide (1.0 M) to regulate pH.

### 2.2. Characterization

We recorded the UV-Vis spectra on a Unico UV-Vis spectrophotometer at 25°C. We performed pH measurements by using a Denver Instrument Model 780 pH meter equipped with a Metrohm glass electrode. TEM images were prepared with Zeiss - EM10 C - 80 kV. We obtained XRD spectra by D8 Advance type (Bruker, Germany), and Fourier-transform infrared spectroscopy (FT-IR) measurements were performed with Spectrum RX1 (Perkin Elmer, 940 Winter Street, Waltham, Massachusetts 02451, USA).

### 2.3. AgNP Preparation

We synthesized AgNP-functionalized *β*-cyclodextrin (*β*-CD) (*β*-CD-AgNPs) by a method mentioned in the literature [20]. Firstly, 20 mL NaOH (0.05 M) solution containing *β*-CD (0.33 g) and then 20 mL AgNO_3_ (0.05 M) solution were added to 60 mL *β*-CD (0.80 g) aqueous solution and was stirred at 500 rpm speed; color of solution quickly turned from colorless to light brown and became cloudy and after overnight, shifted from dark brown to transparent solution.

### 2.4. General Procedure for Preparation of ZDV

In a general procedure, under optimum conditions, we added 300.0 *µ*L of silver nanoparticle (AgNP) aqueous solution to 2.0 mL HCl/NaOH (pH = 2.0) solution followed by adding ZDV to the reaction solution at different concentrations. Also, we recorded the probe solution absorbance at 410 nm and 565 nm.

### 2.5. Preparation of ZDV Tablets

We performed the sample preparation steps to determine ZDV content in the produced tablets according to the reported protocol [24]. Additionally, we ground and mixed ten ZDV tablets (150 mg and 300 mg per tablet) thoroughly. An adequate amount of a tablet was added in 100 ml of distillate water and then, sonicated for 10 minutes. After filtration, 100 *µ*L of the solution was reached to 10.0 mL by adding 0.5 M NaCl solution. The prepared solution was used for ZDV content analysis as a real analytical sample.

## 3. Results and Discussion

### 3.1. *β*-CD-AgNP Characterization

**Figure 1:**
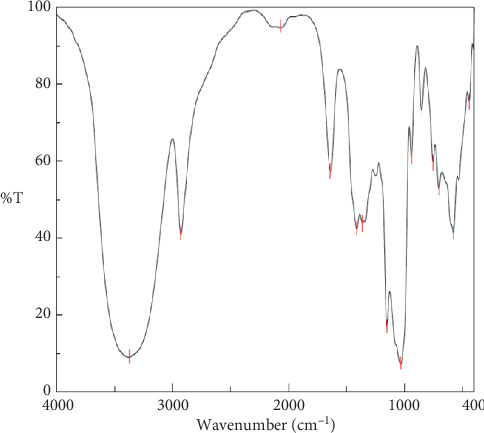
The FT-IR spectra of the AgNPs (a) and *β*-cyclodextrin.

The synthesized AgNPs were fully characterized by TEM, XRD, FT-IR, and UV-Vis spectra.  Figure 1 demonstrates the IR spectra of the AgNPs and *β*-CD. *β*-CD molecule peaks appear at 3417 and 1643 cm^−1^, which were related to the stretching vibration of *β*-CD-OH, the C-H bond stretching vibration results in absorption peaks at 2912 cm^−1^ and 957 cm^−1^, and the appearance of peaks at 758, 701, and 578 cm^−1^ is related to the pyranose ring skeleton vibration (Figure 1(a)); the bands at 1437 cm^−1^ (AgNP-*β*-CD) and 1022 cm^−1^ were attributed to the bending mode of CH_2_ and C-O stretching vibration, respectively [25, 26], and the asymmetric C-O-C stretching vibration of AgNP-*β*-CD and *β*-CD was observed at 1112 cm^−1^ and 1109 cm^−1^, respectively. The spectrum of AgNP-functionalized *β*-CD has a strong IR absorption peak at 1658 cm^−1^(Figure 1), which indicated the oxidation of hydroxyl groups during the reduction process [27]. In comparison with normal vibration wavelength, a little red shift was assigned to the *β*-CD coordination with the AgNP surface [28]. TEM analysis further confirmed the formation of silver nanoparticles.

**Figure 2:**
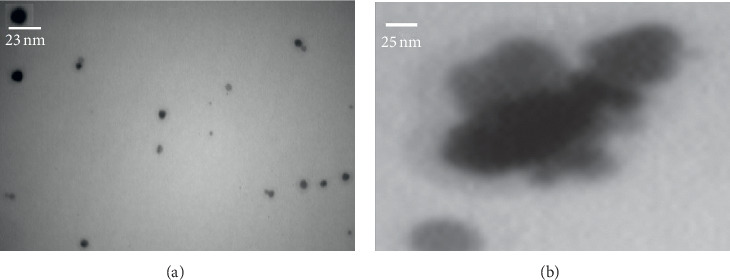
The TEM image analysis of the AgNPs (a) and AgNPs in the presence of zidovudine (b).

The TEM image (Figure 2) shows the uniform dispersed *β*-CD-AgNPs in aqueous solution with an average diameter of 25 nm.

**Figure 3:**
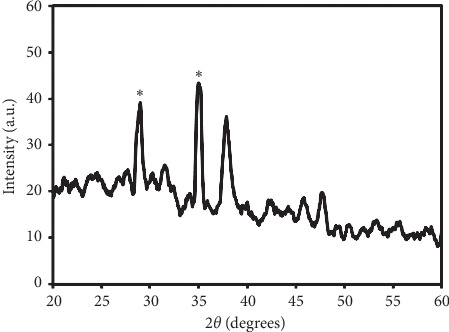
The XRD pattern of AgNPs. Ag metal peaks are shown with asterisk (^∗^).

XRD also characterizes AgNPs were also characterized. As shown in Figure 3, the two peaks at 2*θ* are equal to ∼37.85° and ∼43.6°, which are assigned to the (111) and (200) planes of the face-centered cubic (fcc) silver crystal [29]. The diffraction peaks at 29.1° and 34.7° can be attributed AgNO_3_ residuals [30].

### 3.2. ZDV Colorimetric Detection

**Figure 4:**
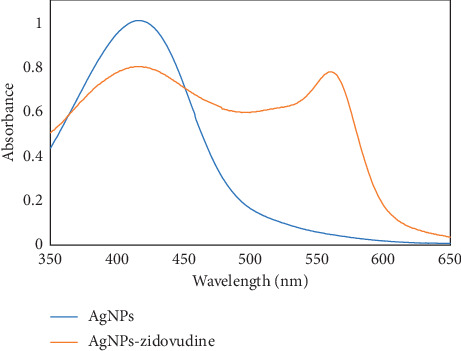
UV-Vis spectra of AgNPs in the absence and presence of zidovudine.

The AgNP UV-Vis spectrum in the absence and presence of ZDV is shown in Figure 4.

**Scheme 2:**
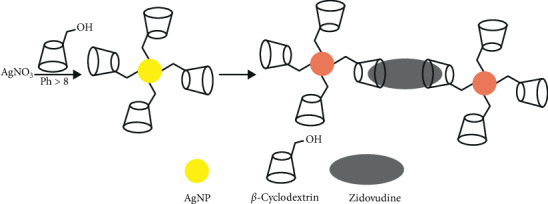
Schematic diagram of calorimetric detection of zidovudine based on *β*-CD and its interaction with AgNPs.

In the presence of ZDV, *β*-CD-AgNPs dramatically change color from brownish-yellow to red, which could be detected by the naked eye easily. In Scheme 2, a novel sensing approach to detect ZDV is introduced.

Based on ZDV concentration, the color and SPR intensity of AgNP solutions were changed gradually. The coordinating interaction of ZDV on the individual AgNP surface with the cavity–OH groups of *β*-CD might cause this shifting. CD molecules have a cavity in which hydroxyl groups are present and interact with the silver nanoparticles through hydroxyl-beta groups on the cavity and cause silver nanoparticle stability [20]. Hydroxyl groups inside a cavitation on a nanoparticle can form hydrogen bonding with other hydroxyl groups, containing hollow groups, and cause aggregation.

### 3.3. Experimental Condition Optimization

#### 3.3.1. Effect of pH

**Figure 5:**
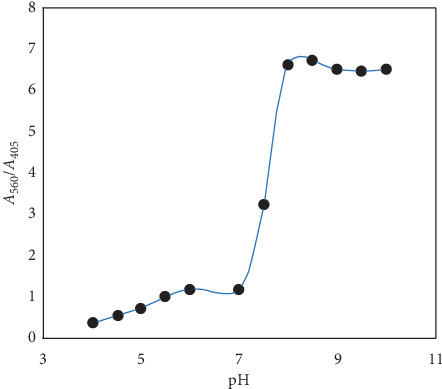
UV-Vis spectra of AgNPs prepared under different pH values. Experimental conditions: 5.0 × 10^−4^ M of zidovudine, pH 4.0–10.0 in HCl/NaOH solution, and response time of 15 min.

Considered to evaluate optimal pH for detecting ZDV with *β*-CD-AgNPs, we utilized UV-Vis spectroscopy of *β*-CD-AgNPs by adding ZDV at different pH ranging from 4.0 to 10.0 (Figure 5). In HCl/NaOH solution, the maximum change (*A*_405_/*A*_560_: absorbance ratio changes) in the optical density was observed at pH 8.0. The prepared particles are unstable at pH below 8.0 due to loss of stability in the form of nanoparticles. Consequently, pH = 8.0 was chosen to adjust ZDV pH solution samples.

#### 3.3.2. Effect of AgNP Concentration

**Figure 6:**
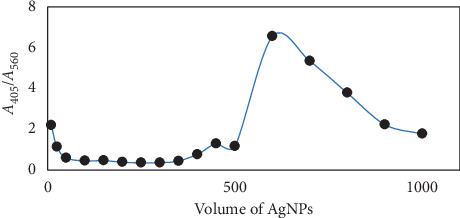
Effect of the volume of AgNPs on the response of the sensor; experimental conditions: 5.0 × 10^−4^ M of zidovudine, pH 8.0 in HCl/NaOH solution, and response time of 15 min.

Clearly, the AgNP concentration could significantly have an effect on the sensitivity and linearity of the method.  Figure 6 shows the *β*-CD-AgNP concentration effects on UV-Vis absorption. We measured the absorbance of a series of solutions including 5.0 × 10^−4^ M ZDV and various concentrations of *β*-CD-AgNPs in HCl/NaOH solution (pH = 8.0) and found the sufficient amount of AgNPs (600.0 *µ*l). Hence, 600.0 *µ*l of AgNP solution was used in further experiments.

#### 3.3.3. Effect of Reaction Time

**Figure 7:**
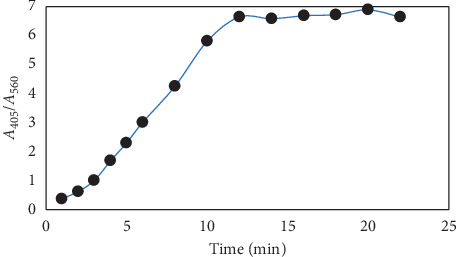
Reaction time of the colorimetric sensor for 5.0 × 10^−4^ zidovudine.

We investigated the incubation time effect on the absorption peak of AgNPs interacting with ZDV. As shown in Figure 7, the maximum differences of the absorption occurred after about 15.0 minutes.

### 3.4. Limit of Detection and Calibration Curve

**Figure 8:**
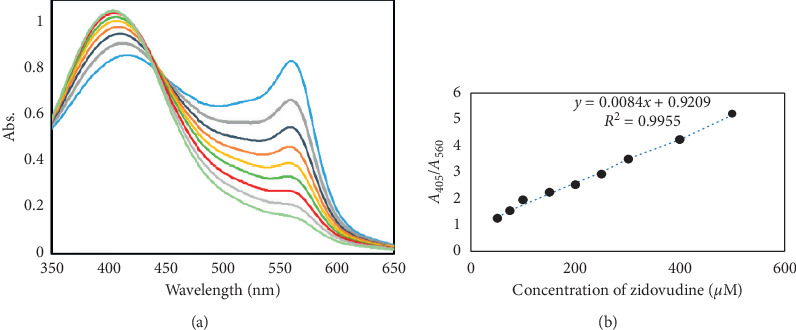
Absorbance peaks and calibration curve for AgNPs in the presence of different concentrations of zidovudine.

Figure 8 shows the *β*-CD-AgNP absorption spectra changes after adding different ZDV concentrations. A linear correlation exists between *Α*_405_/*A*_560_ (absorbance change ratio) and ZDV concentration over a concentration range of 50.0 to 500.0 *µ*M. The calibration equation is Δ*Α* = 0.0084 C (*µ*M) + 0.9209 (*R*^2^ = 0.9955). The detection limit of the presented design was determined from three times and found to be 42 *µ*M.

### 3.5. Repeatability and Reproducibility

To examine repeatability and reproducibility of this method, the absorption spectra of five samples and five sensors were tested under optimum conditions for 200 *µ*M of ZDV. The relative standard deviation (RSD) for 5 measurements and 5 sensors was 3.2% and 3.6%, respectively. This could indicate the desirable repeatability and reproducibility of the developed method for ZDV detection.

### 3.6. Real Sample Analysis

To examine the application of the proposed sensor, quantitative analysis of ZDV in its pharmaceutical products was performed under optimal conditions. According to the data (Table 1), high precision (RSD% = 1.9–3.5%; *N* = 3) and acceptable recovery ranges (97.5–100.75%) showed that the proposed sensor might be suggested as a reliable sensor under laboratory conditions.

## 4. Conclusions

Based on unique properties of AgNPs, such as SPR and ability to form different chemical bonds, we presented a visual colorimetric sensor based on *β*-CD-AgNPs for ZDV detection. This biosensor is not only facile, cost-effective and specific but also shows ideal collodial stability and sensitivity with 42 *µ*M limit of detection. The developed nano-based biosensor could successfully detect ZDV in tablet samples. Also, this approach may be usefull for other drugs and chemical agents detection.

We presented a simple, facile, low-cost and efficient, sensitive ZDV sensor based on distinct *β*-CD-AgNPs color change which can be distinguishing visually. The *β*-CD-AgNPs demonstrated an ideal colloidal stability and this approach possessed a relatively good selectivity lowest detection concentration of 42 *µ*M for ZDV detection.

## Figures and Tables

**Table 1:** Determination of zidovudine in the real sample using AgNPs.

Sample	Added (*µ*M)	Found (*µ*M)	Recovery%	RSD%
Tablet	0	120	—	2.3
	80	198	97.5	1.9
	200	321	100.5	2.8
	400	523	100.75	3.5
